# 

**Published:** 1853-01

**Authors:** 


### INDEX.

A

Abscess, cases of, 45, 101, 213; treatment of mammary, 326
Agnew, D. H., case of foreign substance in intestine, by 346
Alabama State Med. Soc., Proceedings of, 304
Albuminuria, 194
Alison, Dr., on vital affinity, 31
Amaurosis, cases of, 153, 218
American Medical Association, proceedings of, 355, 598; publication of transactions, 536
Aneurism, after venesection, cured by flexing limb, 134
Anti-periodics, new, 719
Appointments to chairs, 661, 662; to hospitals, 710
Arabs, diseases of, 307
Ash tree, infusion of leaves of, in gout and rheumatism, 189
Askew, T. F., case of transposition of viscera, by 682
Asparagus as a remedy for hydrophobia, 669
Association of superintendents of institutions for insane, proceedings of, 400
Astley Cooper prize, awarded to Mr. Gray, 574

B

Bache, Franklin's introd. lect., bibliog. notice of, 167
Bardsley, J. L., knighthood of, 574
Bartlett, on the character and writings of Hippocrates, bibliog. notice of, 166
Bebeerine, sulphate of, as an antiperiodic, 557
Bellingham on diseases of heart, bibliog. notice of, 704
Berlin, medical institutions of, 578
Bernard, on elimination of medicines, 315
Betton, T. F., case of tetanus cured by chloroform, by 680
Biddle, J. B., appointment of in Med. Institute, 122; and in Pa. Med. College, 657
Blood, difference in temperature between arterial and venous, 731
Broma and dietetic cocoa, 464
Bronzed liver, absence of in remittent fever, 669
Brown, G. W., notes of cases, by 409, 692
Brown-Séquard, experimental researches, by 25, 137, 205, 280, 490; tributes to, 120, 306, 432; lectures of, 167
Bubo, syphilitic, new mode of treating, 560
Budd on diseases of liver, bibliog. notice of, 562
Bull's maternal management of children, bibliog. notice of, 653
Burwell on chloroform in midwifery, bibliog. notice of, 655

C

Cæsarian operatiou, case of, 459
Calcutta, mortality in, 574
Cancer, Lebert on, 256
Cannabis indica, use of in trismus nascentium, 783
Carpenter's Physiology, bibliog. notice of, 112
Cataract, cases of, 107, 158, 214
Chapman, N., obituary sketch of, 535; dedication poem addressed to, 571; tributes to, 658, 659
Chloroform, in delirium tremens, 33; deaths from, 174, 769, 770; impending death from, 197; in tetanus, 458; in delirium, 559; in ileus, 604, 716
Cholera, progress of, 307, 573, 574, 575
Chorea, case of, 439
Clinical Reports, by Prof. Gilbert, 42, 96, 150, 213
Clymer, N., resignation of, 235

Cobra de capello, case of bite by, 323
Cock's manual of obstetrics, bibliog. notice of, 654
Collodion, use of in chordee, 539
Condie on diseases of children, bibliog. notice of, 703
Conjuntivitis, case of, 218
Contractility of the veins of the bat's wing, 470
Contusions, cases of, 44, 46, 48, 97, 100, 157
Copaiba and cubebs capsules, 126
Copper, ammonio-sulphate of, 189
Corsets, history of, 239
Cucumber ointment, preparation of, 606
Cystic-sarcoma of testicle, case of, 154
Cysticercus in brain, 539
Cysts, sanguineous pelvic, 127
Case of poisoning by nux vomica, recovery, 790
Coffee, effects of, 787

D

Dailey, E. D., on sulphate of Be-beerine, 557
Deaths, of Dr. George Gregory, 169; Sir. W. Newbigging, 170; Mr. A. Walker, 170; Dr. Jonathan Pereira, 170, 575; Dr. W. G. Horner, 229; Orfila, 308; Dr. Graves, 312; Dr. Chapman, 532; Dr. Beaumont, 576; Dr. Richards, 576; Dr. Caldwell, 577; Dr. W. J. Clark, 663; Dr. J. M. Trudeau, 663; Drs. Nye, Jacobson, Robertson, and Friend, 664; Dr. W. Cochran, 711; Mr. Bransby Cooper, 711
De Boismont's hallucinations, bibl. notice of, 654
Deformity from burn, case of, 49
Delegates to American Medical Association, 168, 304, 434; to State Med. Society, 305
Delirium tremens treated by chloroform, 33
Diabetes, treated by rennet and permanganate of potash, 196; by yeast, 250; on intermitting and senile, 602
Diseases of seamen, Horner on, 543, 607, 688, 735
Disinfectants, 177
Dislocation, cases of, 100, 199, 200, 219, 412, 414, 692, 693
Drake, Dr. Daniel, obituary sketch of, 231
Dubois, appointment of, 307

E

Eaux, de Pagliari, de Rabel, de Hepp, 738
Eczema, case of, 106
Effluvia, influence of, 253
Electricity to promote solution of calculi, 197
Elp hantiasis cured by ligature of femoral artery, 591
Ellis' Med. Formulary, bibliog. notice of, 765
Enchondroma, Prof. Gilbert's operation for case of, 746
Endosmosis as a cause of cathartic action of salts, 186
Enlistment of seamen, Horner on, 416
Epiphora, case of, 219
Ergot in retention of urine, 537
Erythema, case of, 159
Esculapian, notice of, 167
Ether, report on, bibl. notice of, 108
Eustachian tube, muscles of, 266; cases of obstruction of, 330, 626

F

Ferguson's practical surgery, bibl. notice of, 221
Forbes, John, knighthood of, 574
Foreign substances in intestine, case of, 346
Fractures, cases of, 158, 219

G

Gangrene, spontaneous, in glucosuria, 718
Gangrenous metastasis from lungs to brain, 324
Gibbs, O. C., on tubercular meningitis, 144
Gilbert, Prof., clinics by 42, 96, 150 213, 746
Glands, enlarged, case of, 107; salivary, anatomy and physiology of, 204
Glasgow Medical Journal, 307
Gluge's Atlas of Pathological Histology, bibliog. notice of, 226
Gobrecht, W. H., reports by, 42, 96, 150, 213, 746
Graduates, medical, in 1853, 433
Grant, memoir of, by Dr. H. S. Patterson, bibliog. notice of, 118
Growth, non-malignant, re-appearance of after excision, 134; polypoid of heart, case of, 135
Gutta percha, use of in diseases of skin, 442

H

Hæmatocele of spermatic cord, cases of, 331
Hall, Marshall, visit of, 237; lecture by, on zoonomia 269
Hamilton's fracture tables, bibliog. notice of, 655
Hand, inflammation of, case of, 217
Hare-lip, case of, 215
Hazlett, W. B., on treatment of bubo, 560
Headland on actions of medicines, bibliog. notice of, 428
Healthfulness, comparative, of England, France, Prussia, &c., 573
Henle's General Pathology, bibliog. notice of, 649
Henry, Bernard, appointment of to Med. Institute, 122
Hernia, cases of, 46, 101, 218, 409
Hiester, J. P., on influence of mineral kingdom in disease, 407
Hooper's Physician's vade mecum, bibliog. notice of, 654
Horner, G. R. B., on medical topography of Fauquier Co., Va., 346; on enlistment of seaman, 416; on naval hygiene, 475; on diseases of seamen, 543, 607, 688, 735; case of suffocation reported by, 561
Horner, W. E., surgical sketches by 1, 69; obituary notice of, 229; tribute to, 659
Hospital, Berlin Lying-in, 328; puerperal miasm in, 329
Humulus, as an anti-periodic, 718
Hunter on venereal, bibl. notice of, 765
Hydrocele of neck, case of, 220
Hydrophobia, M. Hall on treatment of, 195

I J

Ice, application of in typhus fever, 441
Jenner testimonial, 573
Jewell, Wilson, on the mortality of Philadelphia, 86, 295, 671: report on yellow fever, by, bibliog. notice of, 765
Indigo, on the occurrence of, in human urine, 683
Injuries, extensive results of, 151
Instructions to drug examiners, 712
Iodide of potassium, as an antidote for lead and mercury, 162, 717; a cause of tetanic symptoms, 294
Iodide of sodium in syphilis, 199
Iodine, injections of joints, 198; as a preventive of mammary abscess, 292; after burns, 593
Johns' clinical phrase book, bibliog. notice of, 354
Jones, H. Bence, on animal chemistry, bibliog. notice of, 115
Journals, new notices of, 574
Juniper, compound infusion of, 670
Iron, syrup of sesquinitrate of, 188; sesquichloride of, to produce coagulation of blood in aneurism, 264, 457, 730

K

Kino, new source of, 260
Kirkbride's report of Pa. Hospital, bibliog. notice of, 159

L

Lardner's hand books, bibliog. notice of, 228
Laryngeal affections, lecture on, by Dr. Todd, 240
Leatherman, Jonathan, on delirium tremens treated by chloroform, 33
Leeches, best means of preserving, 188
Lemon-juice, use of in acute rheumatism, 600
Lightning, cases of persons struck by, 177
Lithotomy, recto-urethral operation for,
Little on deformities, bibl. notice of 564
Löwig's organic chemistry, bibliog. notice of, 347
Lucifer matches, case of poisoning by, 541
Lumbago, case of, 117
Lycoperdon proteus, anæsthetic action of, 604

M

Maupin, S., appointment of, 536
Medical Society of Pa., proceedings of, 381
Medical Topography of Fauquier co., Va., 341
Medullary fungus, case of, 104
Meigs, J. F., on diseases of children, bibl. notice of, 632
Melsens on iodide of potassium as an antidote for lead and mercury, bibliog. notice of, 162
Membrana tympani, artificial, 730
Menstruation, effects of, on lactation, 792
Mercury, test for, 185
Miller's practice of surgery, bibl. notice of, 652

Mineral kingdom, on influence of, in disease, 407
Mitchell, J. K., poem by, to Dr. Chapman, 571
Morphia, iodohydrate of, 188
Mortality of Philadelphia, W. Jewell on, 86, 295, 671
Mortification, case of, induced by pregnancy, 267
Murder trial, 122

N

Naval Board, assistant surgeons passed by, 169
Naval hygiene, Horner on, 475
Nævus Maternus, case of, 45
Needle in foot, case of, 44
Neill, John, on a peculiar obstruction of Eustachian tube, 626
Neligan's diseases of skin, bibliog. notice of, 62
New Jersey Med. Institute, notice of, 120
Northern Med. Association election of officers, by 120
Norwalk catastrophe, account of, 380

O

Oil of ginger, 465
Oleum morrhuæ, 189
Opium trade, 235
Orchitis, case of, 159
Oxalate of lime, in relation to neuralgia, 598
Oxalic acid, generation of, in syrup of sesquinitrate of iron, 189

P

Paget's Surgical Pathology, bibl. notice of, 759
Palate, cleft, case of, 106
Paracentesis thoracis, case of, 697
Paralysis, case of, 99
Paris, state of profession in, 169
" health of, 238
" typhus fever in, 307
" academie de medicine of, 307
Parotitis, case of, 219
Patterson, H. S., on oil of pumkin seeds in tape-worm, 629
" resignation of, 657
Pharmaceutical Association, proceedings of, bibl. notice of, 707
Pharyngitis, case of, 96
Phillips, D. B., on tubercular consumption, 37
" on iodide of potassium, 294
Phlegmasia dolens, on pathology of, 460
Phosphate of lime in relation to nutrition 197
Physicians, duties and labors of, 431
" for emigrant ships, necessity of, 431
" visiting list for 1854, bibl. notice of, 656
Pneumonia, on pathological physiology of, 190
Polypus, rare case of, 53
Poisoning by atropia applied to the conjunctiva, 790
Pope, H. J., on chloroform for relief of delirium, 559
Porrigo favosa, case of, 157
Precocious development of sexual system, case of, 745
Prescriber's Pharmacopœia, bibl. notice of, 655
Ptosis, case of, 102
Pumpkin seeds, oil of, as remedy for tape-worm, 629

Q

Quackery in Paris, 433
Queen's college for ladies, 236
Quinine, sulphate of, adulteration of, 185
" hospital sulphate of, 187
" iodohydrate of, 188
" treatment of rheumatism, by 438
" " of yellow fever, by 664

R

Reese, J. J., introd. lect., bibliog. notice of, 167
Regnault's chemistry, bibl. notice of, 425
Resection of superior maxillary bones, cases of, 595
Respiration, state of, in pressure on brain, 194
" in connection with causes of consumption, 319
Rhubarb, new test for, 262
" imperial, 721
Ricord's letters on syphilis, bibliog. notice of, 303
Rogers, James B., memoir of, by Dr. Carson, bibliogr. notice of, 67
Ruta graveolens, case of poisoning from 720

S

Savannah Medical College, 710
Scirrhus of the uterus, case of, 127
Scurvy, acids in, 124
Semen, composition of, in old men 469
Silliman's Chemistry, bibliog. notice of, 117
Simon's General Pathology, bibliog. notice of, 119
Sinus, case of, 103, 157
Sloane, Sir Hans, notice of, 176
Smith, H. H.'s, system of operative surgery, bibliog. notice of, 54
Smith, Joseph, on oil of turpentine in puerperal tympanites, 147
Southern Journal, notice of, 167
Sperino on syphilisation, bibliog. notice of, 752
Spinal irritation, case of, 104, 150, 216
Stewart, H. C., on iodine as a preventive of mammary abscess, 292
Stoakley, W. S., on dormant vitality of tobacco seed, 630
Strabismus, cases of, 100, 105, 151, 217
" new mode of operating in, 593
Strychnine, case of poisoning from, relieved by chloroform, 337
Suffocation, case of, 561
Sulphuric acid, case of suffocation by, 319

T

Tannic acid, on preparation of, 466
Taraxacum juice, preparation of, 605
Taylor's Medical Jurisprudence, bibl. notice of, 564
Teas, black and green, 466
Teleangiectasy, case of, 42
Temperature of body, laws regulating, 202
Tennessee Med. College, transactions of, bibl. notice of, 656
Tents, sponge, 266
Tetanus, cases of cured by chloroform, 458, 680
Tilt on female hygiene, bibl. notice of, 563
Tinnitus aurium, case of, 100
Tobacco seed, dormant vitality of, 630
Toe-nail inverted, case of, 214
Tracheotomy, in epilepsy, debate on, 448
" in croup, case of, 455
" for extraction of a piece of glass, 593
Transposition of viscera, case of 682
Trismus nascentium, two cases of, which recovered under the use of cannabis indica, 783
Tubercle, Virchow on, 192
Tubercular consumption, D. B. Phillips on, 37
" meningitis, O. C. Gibbs on, 144
Tumors, cases of, 53, 99, 152
Turpentine, oil of in puerperal tympanites, 147
" in yellow fever, 668
Typhoid fever, treatment of, in Paris, 437

U

Ulcer, varicose, case of, 97
University of Virginia, resignations in, 305
Upshur, G. L., case of poisoning from strychnine, by, 337
" case of occlusion of vagina, by 523
Urea, reagent to detect quantity of, in urine, 185, 733
Urine, silicious deposits in, 193
Uterine fibres, composition of, in pregnancy, 204
Uterus, anteflexion of in pregnancy, 728
Uva ursi, as a substitute for ergot, 727

V

Vaccination act in Great Britain, 711
Vagina, congenital occlusion of, cases of, 132, 523
" distention of after parturition, 540
Varus, cases of, 45, 52, 102, 150
Veratria, treatment of rheumatism, by, 438
Viability, early, cases of, 597
Virginia Medical and Surgical Journal, notice of, 121
Vitiligooidea, case of, 258

W

Walton's ophthalmic surgery, bibl. notice of, 643
What to observe at the bedside, bibliog. notice of, 166
Wilde's Aural Surgery, bibl. notice of, 636
Williams' principles of medicine, bibl. notice of, 564
Wilson, G. A., appointment of, 536
Wounds, cases of, 98, 695

Wythes' pocket dose book, bibliog. notice of, 166
" Microscopist, bibl. notice of; 657

Y

Yaws, notice of, 718
Yellow fever in Philadelphia, in 1853, account of, 567, 708
" in New Orleans, 664
" British Quarantine report on, bibl. notice of, 700

Z

Zinc, chloride of, case of poisoning from, 722
Zinc ointment, readiness with which it becomes rancid, 187
Zoonmonia, lecture on, by Dr. M. Hall, 269

